# Role of finite element analysis for selection of single point fixation in zygomaticomaxillary complex fracture

**DOI:** 10.1186/s12903-023-03822-1

**Published:** 2024-01-04

**Authors:** Shaimaa Mohsen Refahee, Mahmoud Elsayed Khalifa, Mohamed Gamal Askar, Maram N. Breshah

**Affiliations:** 1 Oral & Maxillofacial Surgery Department, Faculty of Dentistry, Fayoum University, Fayoum, Egypt; 2 Oral & Maxillofacial Surgery Department, Faculty of Dentistry, Tanta University, Tanta, Egypt; 3 Mechanical Power Engineering Department, Faculty of Engineering, Helwan University, Cairo, Egypt

**Keywords:** Finite element analysis, Stability, Zygoma, Zygomaticomaxillary complex, Fixation

## Abstract

**Background:**

One-point fixation was superior to the two and three-points fixation in minimally displaced zygomaticomaxillary complex (ZMC) fracture regarding the cost, invasiveness, scaring, number of wounds, and operation time. Accordingly, this study aimed to predict which one-point fixation is the most stable in managing minimally displaced ZMC fracture.

**Material & methods:**

This study simulated the different one-point fixation approaches on three ZMC models after fracture reduction and application of all forces exerted on the fractured area. The findings were represented as stress impact on the ZMC fracture and plating system as well as the inter-fragments micro-motion.

**Results:**

The von misses stresses of plates for the zygomaticofrontal, infra-orbital rim, and zygomaticomaxillary buttress model were (66.508, 1.285, and1.16 MPa) respectively. While the screws’ von misses for the infraorbital rim, zygomaticofrontal, and zygomaticomaxillary buttress models were (13.8, 4.05, and 1.60 MPa) respectively. Whereas, the maximum principles stress at zygomaticofrontal, zygomaticomaxillary buttress, and infraorbital rim models were (37.03, 37.01, and 34.46 MPa) respectively. In addition, the inter-fragment micro-motion for zygomaticomaxillary buttress, infraorbital rim, and zygomaticofrontal models were (0.26, 0.25, and 0.15 mm) respectively.

**Conclusion:**

One-point fixation at zygomaticomaxillary buttress is the preferred point because it is exposed to low stresses, and the inter-fragment micro-motion is within the approved limit with the elements in the same direction of fixation which indicates the rigid fixation. In addition, it is less palpable and scarless.

**Trial registration:**

clinical trial.gov (NCT05819372) at 19/04/2023.

## Introduction

The zygomatico-maxillary complex (ZMC) is the major middle face component. It supports the mid-face width, the orbital content, and the malar eminence projection anteriorly [1].

It consists of thick bone vertical buttresses that transfer and distribute the forces from the teeth-bearing area to the cranium as nasomaxillary, zygomaticomaxillary, and pterygomaxillary buttresses. These vertical buttresses resist the compressive stress resulting from the trauma. They are supported by other horizontal buttresses that resist the lateral force causing buckling of the facial bone as supraorbital, infraorbital, and alveolar buttresses. Most of the load is transferred by the zygomaticomaxillary buttress, while the inferior orbital rim (IOR) is loaded with minimal stress so it was not necessary to plate this area [2, 3]. Accordingly, ZMC plays a significant role in supporting the facial structure, function, and aesthetic [1].

The main target for ZMC fracture management is restoring the function, stability, and aesthetic of the mid-face and surroundings [4]. Open reduction and internal fixation is the most commonly used approach to achieve this target in the ZMC fracture management [4]. Different plates’ materials, shapes, and dimensions were used for internal fixation of the ZMC fracture. Fixation with 1.5 mm curved titanium mini-plates is the most preferred plate for non-comminuted ZMC fracture fixation at IOR and zygomaticofrontal (ZF) with 2 screws on each side of the fracture line. A 2 mm L-shaped titanium mini plate was used at the zygomaticomaxillary buttress to resist the masseter muscle’s action [5]. Titanium plates are biocompatible, easily adapted, and manipulated. In addition, it is hard enough to give stability to the reduced fracture segments [6, 7]. However, titanium plates need to be removed over the time as they may cause discomfort to the patient and infection. Some studies suggested the use of resorbable plates and screws to avoid the titanium plates’ drawbacks, but their strength is debatable [7–9]. Accordingly, small resorbable microplates can be used at IOR to avoid its palpability under thin skin, as it is considered hard enough with the low values of stress at IOR [10]. The method of fixation depends on the impact of trauma, as it determines the degree of post-fixation segment stability. The ZMC fracture is classified according to the intensity of trauma into low, medium, or high energy patterns. Whereas the low energy pattern described the non or minimally displaced simple isolated fracture with minimally displaced IOR, no orbital content changes, no ocular problem, and no step-off at any part of ZMC. While the high-energy one described the completely displaced/ comminuted ZMC fracture [11]. Different methods of fixation were included in ZMC fracture management as three-, two-, or one-point fixation methods [12–16]. Nasr WF et al. [17] proved that two- and three-point fixation methods had the same results regarding segment stability with a low-cost two-point method in the medium energy pattern of ZMC fracture. But Kim JH et al. and Neto RM et al. [15, 18, 19] supported that one-point fixation was superior to the two and three-point fixation in the low energy pattern of ZMC fracture with intact IOR regarding the cost, invasiveness, scarring, number of wounds, and operation time. One point fixation method was reported to be enough in the low energy pattern of ZMC fracture as Dal Santo et al. [20] proved that the force of masseter muscle was reduced at the fracture side for 4 to 6 months and the cause of post-fixation instability improper pre-surgical segment reduction. In addition, ZMC is not considered to function all the time as the mandible. So, not all separated articulations needs to be fixed. Different studies confirmed that the one-point fixation either at the ZF suture or IOR was not preferred as plates in these sites are palpable, the incision causes a visible scar, and the ZF suture is exposed to high stresses which may lead to hardware failure. In addition, the thin overlaying soft tissue is prone to injury due to penetration by the plates. Accordingly, the used plates are very thin and endanger the rigidity of fixation [14, 21, 22]. Whereas, the zygomaticomaxillary (ZM) buttress was considered the preferred area for fixation because it is a bone buttress that resists muscle force and its incision is scarless [14, 15, 23].

In contrast, Hwang [13] proved that the one-point fixation method at the ZF suture in the management of minimally displaced ZMC fracture can be used. Al-Qattan M and Gelidan A [24] suggested that only one-point fixation at the IOR is effective in minimally displaced medially rotated ZMC fracture. Only one study compared the different one-point fixation methods using three-dimensional (3D) finite element analysis (FEA) and demonstrated that there were no differences concerning segment stability [25]. However, it did not use the plates’ design as used during the surgery. In addition, it did not evaluate the effect of all muscles’ force exerted on the fractured ZMC segment. As masticatory, facial expression muscles that were attached to the ZMC and the biting force exerted bending and shear forces on the fractured segments and affected their instability [26].

The authors hypothesize that each area for the one-point fixation method (either ZF, ZM, or IOR) may have different stresses which may affect the stability after fracture fixation. Accordingly, this recent study aimed to predict which one-point fixation approach is most sufficient and stable in managing minimally displaced ZMC fracture after evaluating all muscles’ forces exerted on it.

## Methods

This FEA study was completed in the oral and maxillofacial department between February and July 2023. It was consented by the research ethics committee, Faculty of Dentistry, Tanta University on February 2023 (#R-os-2-23-3), and documented on the clinical trial. gov (NCT05819372) on April 19, 2023.

As part of this study, multislice computed tomography imaging (CT) (scan parameters 120 KV, 100 Ma, scan time 0.5 s, slice thickness 0.625 mm, and resolution from 226 to 3071HU) with isolated minimally displaced ZMC fractures with no orbital floor fracture, no ocular problem, and no orbital content changes was used to reconstruct three dimensional (3D) finite element models. The fracture was analyzed accurately using Mimics 19.0, EXOCAD, SolidWorks, and ANSYS 19.2. software to predict which point of fixation is valuable.

### Reconstruction of ZMC models

**Fig. 1:**
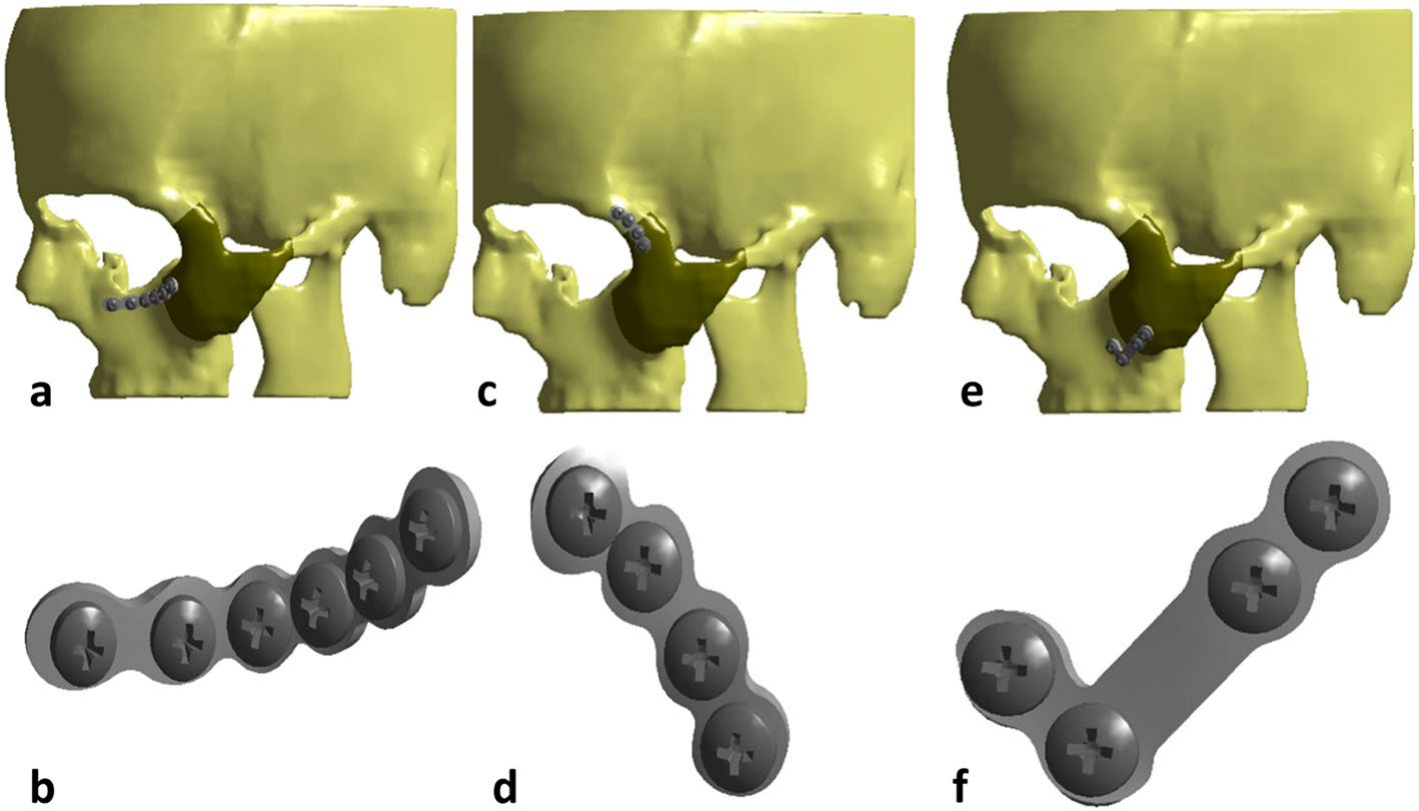
**a** Showing of IOR model. **b** Showing simulation of the mini-plate as used clinically at IOR model. **c** Showing of ZF model. **d** Showing simulation of the mini-plate as used clinically at ZF model. **e** Showing of ZM model. **f** Showing simulation of the mini-plate as used clinically at ZM model

Three 3D finite models were reconstructed by the mirror function of Mimics software based on the DICOM file of CT images to obtain the ZMC bone geometry after reduction. Five fracture lines were defined using the reverse engineering and segmentation function of solidwork software including the inferior orbital rim (IOR); zygomatic frontal suture (ZF); the zygomatic maxillary suture (ZM); the zygomatic arch (ZA) and the zygomatic sphenoidal suture (ZS). Finally, an internal fixation titanium screw-plate system that was identical to that used in the surgery was designed by solidwork software and placed on each model at IOR, ZF, or ZM using ANSYS as follows (Fig. 1a-f). The screw plating system was used with a screw 2 mm in diameter, 4 mm in length, and a plate thickness of 1.5- 2 mm.

### Identification of simulated material properties and mesh creation

According to the values described previously, the mechanical properties of each component (cortical bone, cancellous bone, gingiva, and titanium plating-screw system) were identified in the software [27–32]. Regarding the mesh, a simple unstructured tetrahedral mesh generation was performed with a variable density. The density was lower than 0.2 mm element size around the screws-bone connection and higher away from the interest, with a global element size of 0.9 mm, a tolerance value of 0.045, an aspect ratio of 6.7, skewness of 0.68, and total element and node's numbers listed in (Table 1).

**Table 1:** showing the elements and nodes number of the mesh in each model

Model	Element	Node
IOR	431,582	793,957
ZF	438,457	794,587
ZM	421,459	797,854

*IOR* Infraorbital rim, *ZF *Zygomaticofrontal, *ZM *Zygomaticomaxillary

### Defining the boundary and loading simulation

The boundary condition and load configurations were constant across the finite models. The all model’s boundaries were considered immovable to mimic the surrounding of ZMC fracture with the median occlusion [33]. Frictional contact was set between the plate and bone or the retaining screw in all models with a frictional coefficient of 0.3220 [34].

**Fig. 2:**
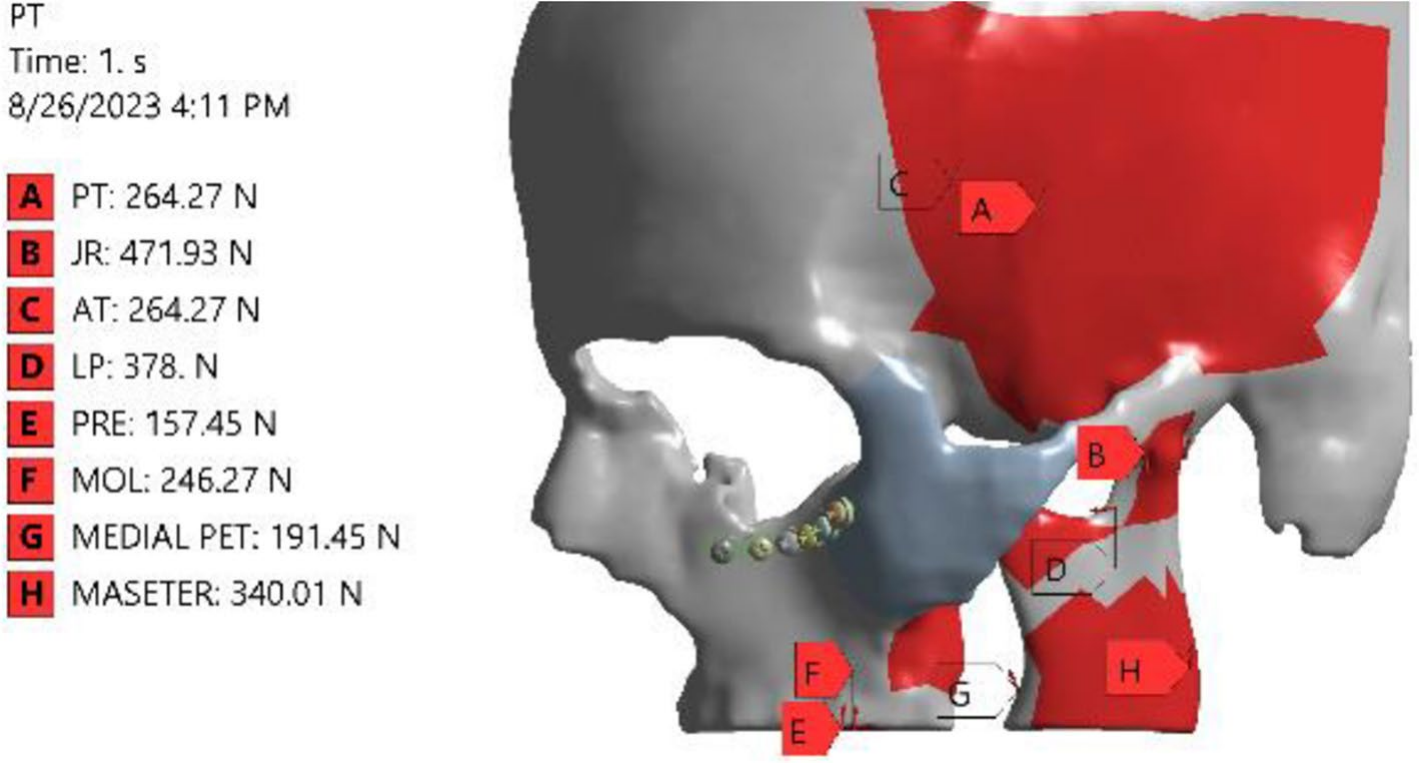
Showing all forces that act on the fractured ZMC area. PT: Posterior temporalis muscles; JR: Joint reaction; AT: Anterior temporalis muscle; LP: lateral pterygoid muscle; MEDIAL PET: medial pterygoid muscle; MASETER: masseter muscle; PRE: biting force (at second premolar); MOL: biting force (at first molar)

All the screws were tightened to the mini-plates with a 30 Ncm tightening torque. The sum of the muscles including the masseter, temporalis, medial, and lateral pterygoids as well as the biting and joint reaction forces that impact the fractured bone were identified in the software as mentioned before [35] and represented in (Fig. 2). Each model’s displacement was prevented using a fixed restraint on the skull inferior border.

### Outcome assessment and data analysis

The FEA revealed stresses at every node in each model. The findings were represented as stress impacts on the ZMC fracture and plate-screw system as well as the displacement between the fracture fragments.

Stress in this study represented by Von Mises and Maximum Principal Stress. Von Mises stress was used to determine which stress value would cause the failure of a screw-plating system. While Maximum Principal Stress indicates the value of the stress that affects the bone around the fracture line and causes bone resorption if it exceeds 50Mpa [36]. In addition, the displacement between the two bony fragments relative to the surrounding zone was analyzed which is an indicator of fixation rigidity and fracture stability.

The numeric data was transformed into color graphics. The colors ranged from red, which represents the maximum stress to blue, which represents the minimal stress.

## Results

### Von misses stresses for all models

**Fig. 3:**
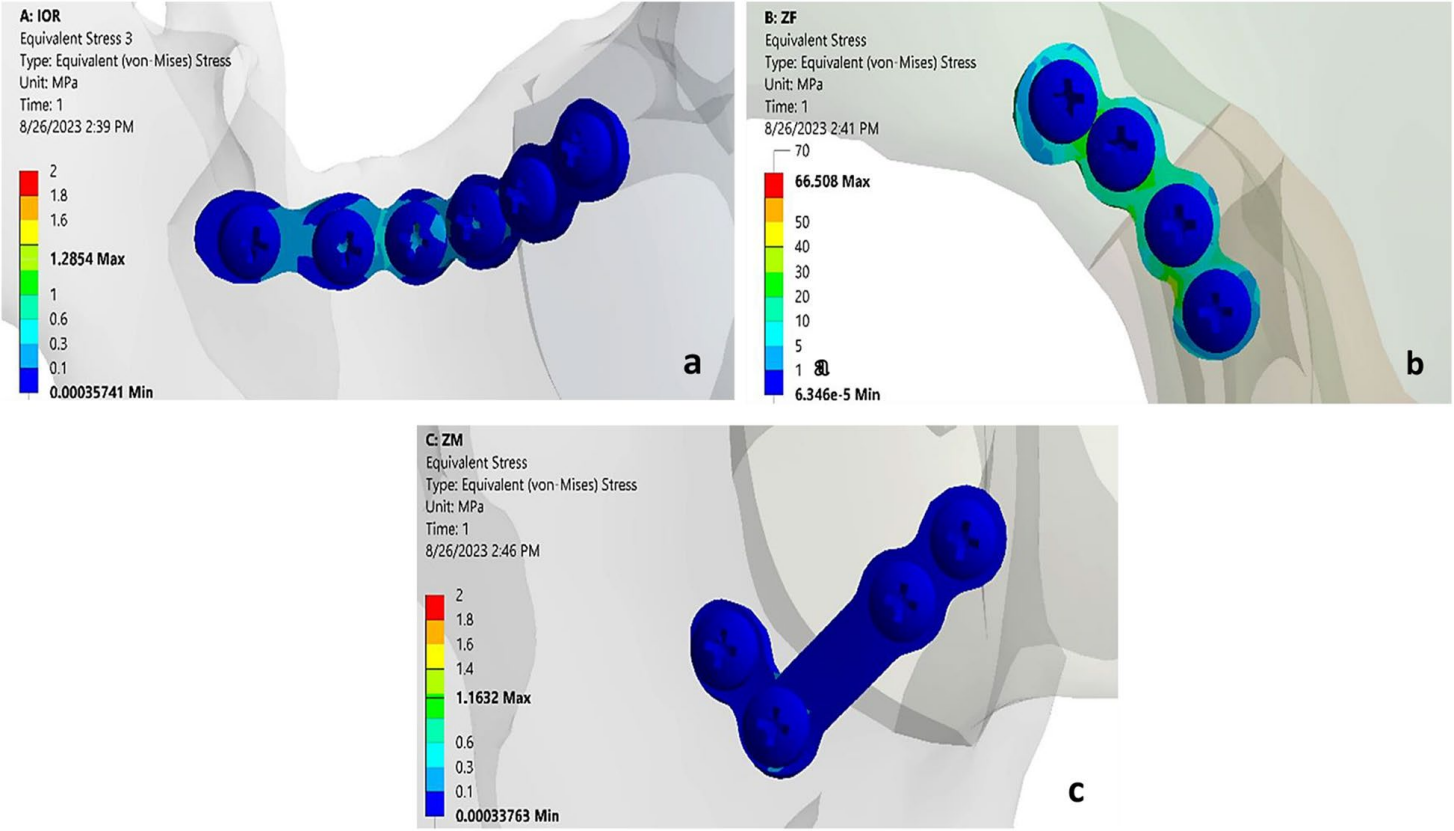
**a** Showing von Misses stress on the plate of the IOR model. **b** Showing von Misses stress on the plate of the ZF model. **c** Showing von Misses stress on the plate of the ZM model

Regarding the mini- plates’ von misses stresses, the maximum von misses stress (66.508 MPa) was recorded for the ZF model, followed by the IOR model (1.285 MPa), whereas the lowest stresses were recorded for the ZM model’s plate (1.16 MPa) (Fig. 3a-c).

**Fig. 4:**
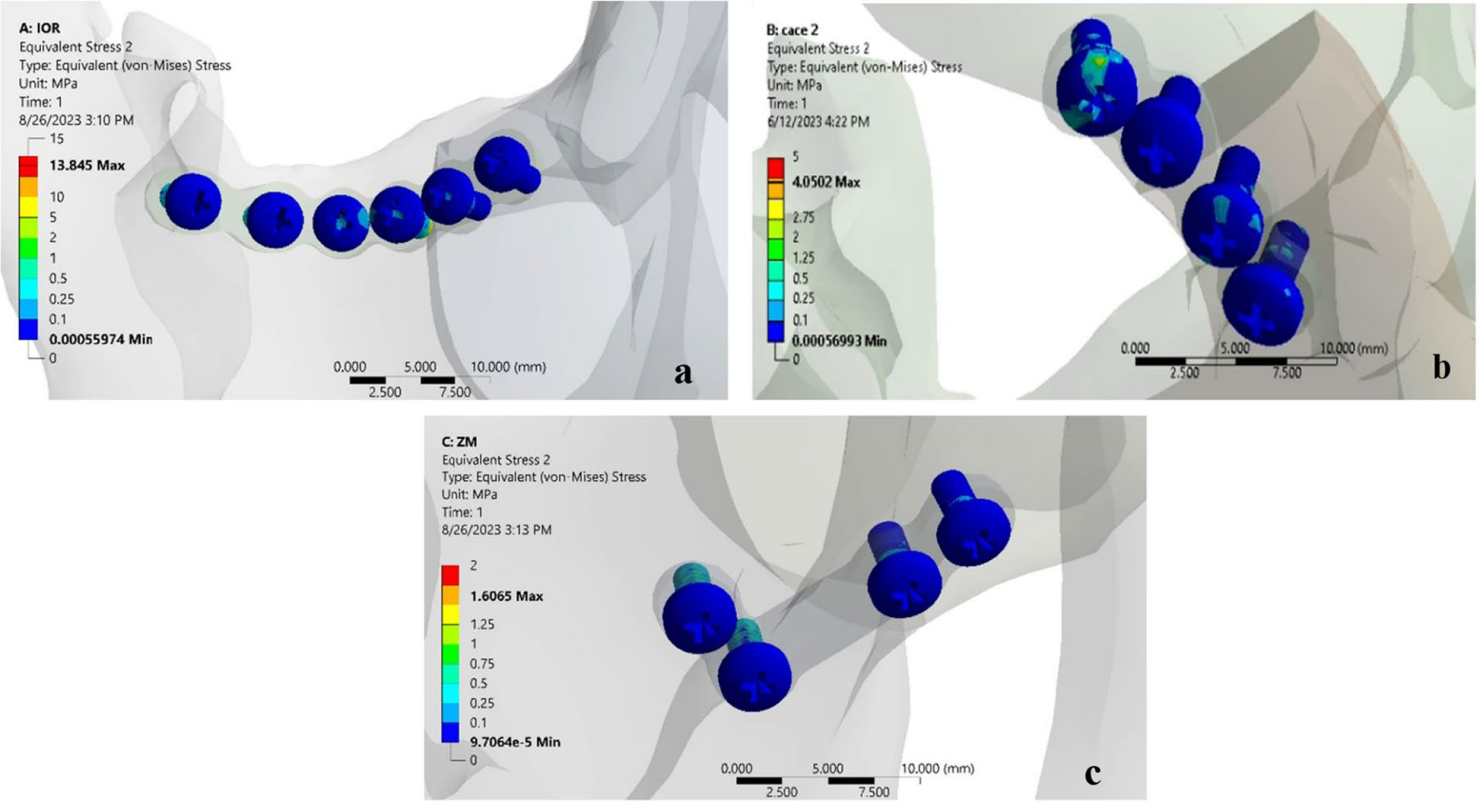
**a** Showing von Misses stress on the screws of the IOR model. **b** Showing von Misses stress on the screws of the ZF model. **c** Showing von Misses stress on the screws of the ZM model

Regarding screws’ von misses stress, the maximum one was recorded for the IOR model’s screws (13.8 MPa), followed by the ZF model’s screws (4.05 MPa). While the lowest stress was recorded for the ZM model’s screws (1.60 MPa) (Fig. 4a-c).

### Maximum principal stresses of the bone

**Fig. 5:**
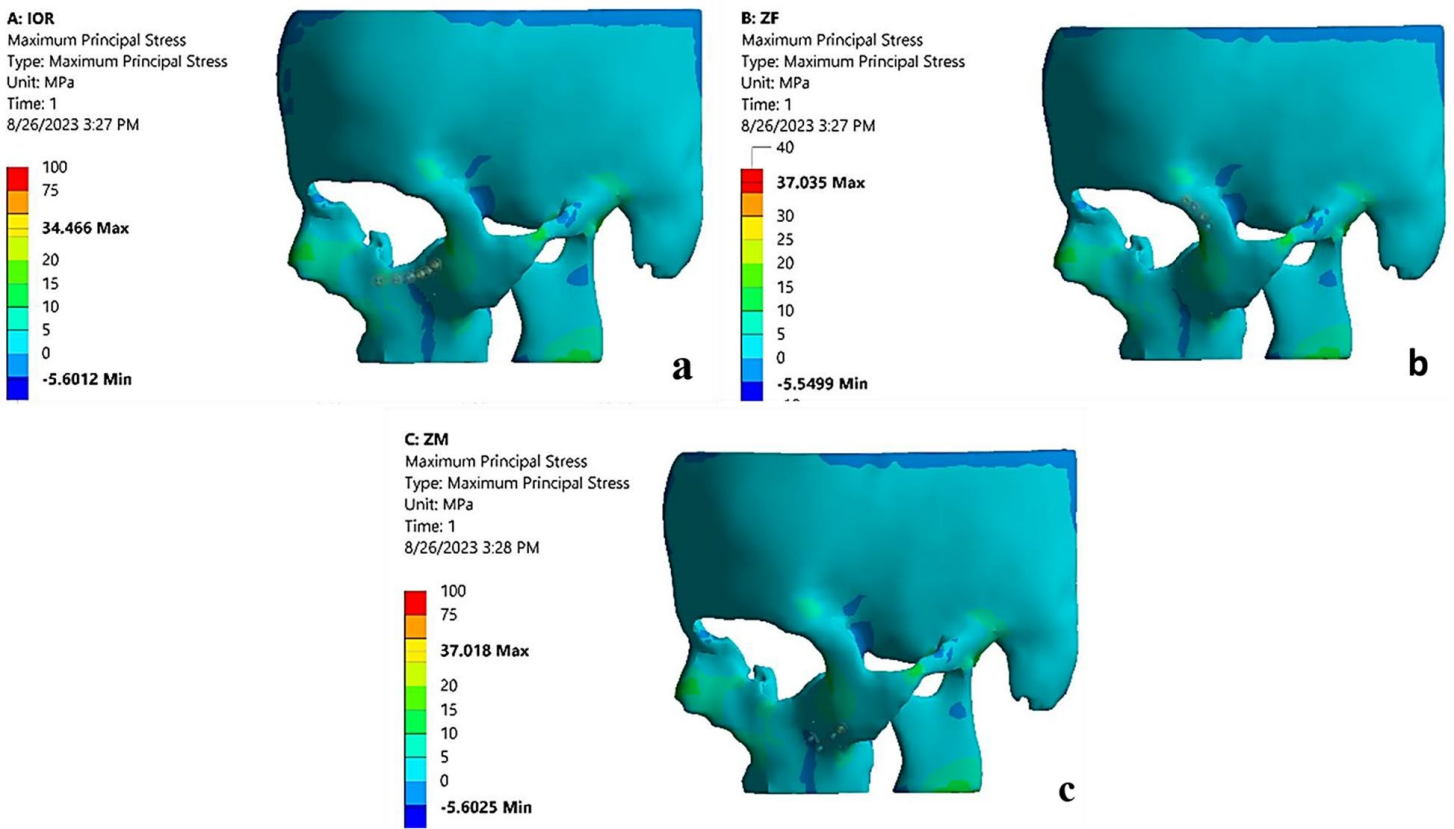
**a** Showing maximum principal stress on the bone of the IOR model. **b** Showing maximum principal stress on the bone of the ZF model. **c** Showing maximum principal stress on the bone of the ZM model

Regarding maximum principal stress, the highest one (37.035 MPa) was recorded for the ZF model, followed by the ZM model (37.018 MPa). While, the lowest one was recorded for the IOR model (34.46 MPa) (Fig. 5a-c).

### Micromotions for all models

**Fig. 6:**
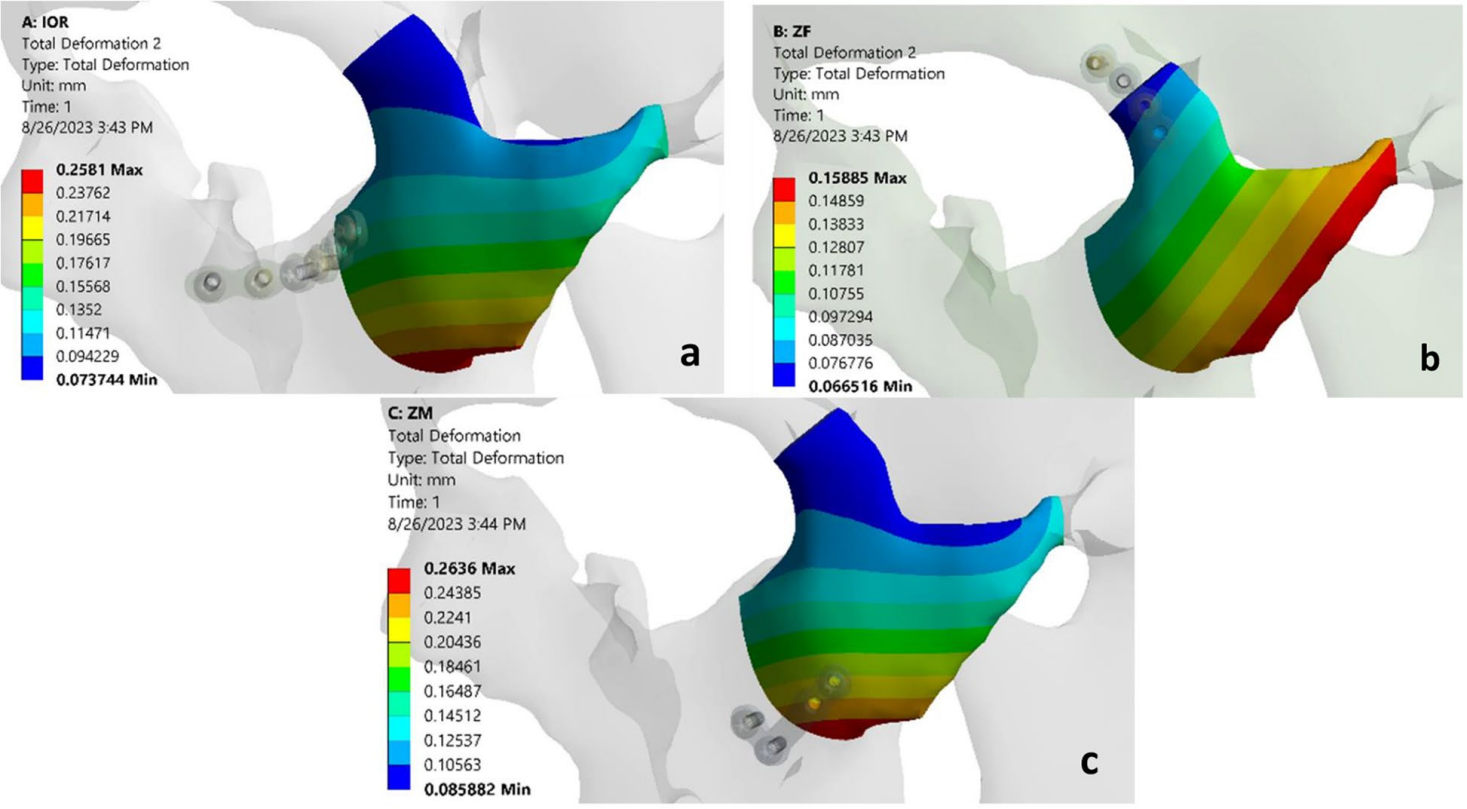
**a** Showing the interfragments micromotion at IOR model. **b** Showing the interfragments micromotion at ZF model. **c** Showing the interfragments micromotion at ZM model

Regarding the interfragment micromotion, the maximum one (0.26 mm) was recorded for the ZM model, followed by the IOR model micromotions (0.25 mm). While, the lowest one was recorded for the ZF model (0.15 mm) (Fig. 6a-c). In addition, elements were orthogonally recorded on the fixation at ZF. On the other hand, elements were recorded along the same direction of the fracture line at both IOR and ZM.

## Discussion

Zygomaticomaxillary complex fractures are among the most common maxillofacial trauma and their management is considered challenging. Open reduction and internal fixation is considered the standard method for treating ZMC fractures [4]. Multiple fixation approaches have been used according to the fracture severity and extension as one-, two-, and three-point fixation [12, 16].

Because of growing concern about scaring, minimally invasive procedures were used by surgeons to treat ZMC fractures using one-point fixation. One-point fixation is superior to the other fixation approach in the case of a minimally displaced ZMC fracture in the terms of cost, invasiveness, scarring, number of wounds, consequent infection, and operation time [13, 15, 18, 23].

The authors hypothesize that each area for the one-point fixation method (either ZF, ZM, or IOR) may have different stresses which may affect the stability after fracture fixation. Accordingly, the specific aim of the study was to predict which one-point fixation approach is the most sufficient and stable in the fixation of minimally displaced ZMC fracture after evaluation of all forces exerted on it.

In this study, the 3D-FEA was used for diagraming the stress distribution over the IOR, ZF, and ZM to predict the most stable one-point fixation approach under simulated muscles’ forces for cases of minimally displaced ZMC fracture. The FEA had been used to analyze the facial structures, and provide fine details about the stress distribution and displacements [37, 38].

According to Ben-Nissan, all forces that exert a load on the fractured area were simulated in this FEA study as even a small muscle force may cause displacement of the fractured bone fragment. In contrast, Fallahi et al. [25] simulated the force of the masseter and pterygoid muscles only with 125 N force on the fractured ZMC.

Regarding the Von Misses stress on the titanium plate, it was used to determine which stress value would cause the failure of a screw-plating system. The maximum von Misses stress (66.508 MPa) was documented for the ZF model, followed by the IOR model (1.285 MPa), and the lowest stresses were recorded for the ZM model’s plate (1.16 MPa). However, these results are not significant because all values were lower than the plate’s fatigue limit (900–1000 MPa) and yield stress (934 MPa). Accordingly, the plates at the different sites were not expected to fail [3, 39].

About the maximum principal stress recorded for the bone surrounding the screw-plating system, the highest maximum principal stress (37.035 MPa) was recorded for the ZF model, followed by the ZM model (37.018 MPa), and the lowest one was recorded for the IOR model (34.46 MPa). These stress values were consistent with Hanemann et al. and Hart NH et al. [10, 40] who proved that the ZF fixation point exposed to high stress could cause a plate-screw system failure in comparison to the ZM fixation point that was exposed to the least stress. All maximum principal stress values in this FEA study were considered clinically insignificant since they were below 50 MPa. This conclusion was consistent with Sugiura et al. [36] who reported that if the maximum principal stress of the bone surrounding the screw exceeds 50 MPa, it may cause bone resorption.

Concerning the interfragment micromotion, the one (0.263 mm) was recorded for the ZM model, followed by the IOR model (0.257 mm). Then the lowest one was recorded for the ZF model (0.158 mm). The micromotion between the two fracture fragments affects the fracture healing. All micromotion values were observed between 0.15 and 0.50 mm with no significance that indicated a single fixation point was adequate for post-fixation stability and did not affect bone healing [41]. This was in agreement with Fallahia et al. [25] who used finite element analysis to study the effects of different plate fixation methods in the zygomaticomaxillary complex and observed no significance between IOR, ZF, and ZM regarding fracture stability.

The fixation rigidity also depends on the direction of the elements in relation to the fracture line. This study recorded the orthogonal direction of the elements to the fracture line at the ZF area which represents the behavior of shear loading on the fixation plates and causes less fixation stability that can affect bone healing. On the other hand elements were recorded along the same direction of fixation at both IOR and ZM which represents the behavior of tensile and compression loading that indicates more fixation stability. These results were in agreement with Prado FB et al. [42]. The strengths of this study were the use of FEA with a simulation of all forces exerted on the fractured area as the small muscle contraction affects the stability of the fractured segments. In addition, the plates’ geometry was simulated as used in the surgical field with a semi-circular plate at IOR, a straight mini plate at ZF, and an L-shaped plate at ZM, to simulate the clinical environment. The main drawback of this study was the simulation of bone as a homogenous and isotropic material when in fact it is heterogeneous and anisotropic. This is done in most FEA studies for simplicity. However, simulating the bone as heterogeneous and anisotropic makes it so tough to simulate rather than the huge time it takes. Furthermore, this method of simulation did not affect the accuracy of the results.

The clinical significance of this study is confined to the ability to use a one-point fixation method for the simple ZMC minimal displaced fracture with no ocular problem or orbital volume changes as it respects the soft tissue, decreases the operating time, infection susceptibility, cost, and does not affect bone healing. The ZM is considered the best choice for one-point fixation. It provides more rigid fixation and stable fracture as the stress and the micromotion values at ZM were within the approved limit with parallel elements’ movement to the fracture line (tensile/ compression loading). In addition, it is less palpable and scarless as the incision was made intraorally.

Further studies comparing the one-point fixation at ZM, ZF, and IOR with large a sample size and different fracture patterns are recommended.

## Conclusion

According to the results of the study, the authors suggested ZM as the most preferred one-point fixation approach for ZMC minimally displaced fracture management. It provides more stable fracture and rigid fixation as stress and micromotion values at ZM were within the approved limit with parallel elements’ movement to the fracture line (tensile/ compression loading). In addition, it is less palpable and scarless as the incision was made intraorally.

## Data Availability

All data are available with the corresponding author.

## Abbreviations

ZMC: Zygomaticomaxillary complex
ZM: Zygomaticomaxillary
IOR: Inferior orbital rim
ZF: Zygomaticofrontal
FEA: Finite elements analysis
CT: Computed tomography imaging
ZS: Zygomatic sphenoidal suture
ZA: Zygomatic arch
DICOM: Digital Imaging and Communications in Medicine

## References

[1] Ribeiro MC, Regalo SCH, Pepato AO, Siéssere S, de Souza LG, Sverzut CE (2011). Bite force, electromyography, and mandible mobility during the 6-month period after surgical treatment for isolated fractures of the zygomatico-orbital complex. Oral Surg Oral Med Oral Pathol Oral Radiol Endod.

[2] Le FOrt R (1901). Etude experimentale sur les fractures de la machoire superieure. Revue Chirurgio.

[3] Wroe S, Ferrara TL, McHenry CR, Curnoe D, Chamoli U. The craniomandibular mechanics of being human. Proc Biol Sci. 2010;277(1700):3579–3586. 10.1098/rspb.2010.0509 PMC2982237 20554545

[4] Yonehara Y, Hirabayashi S, Tachi M, Ishii H (2005). Treatment of zygomatic fractures without inferior orbital rim fixation. J Craniofac Surg.

[5] Al-Watary MQ, Gao H, Song L (2023). Stability of different fixation methods after reduction malarplasty under average and maximum masticatory forces: a finite element analysis. BioMed Eng OnLine.

[6] Kasrai LA, Hearn TA, Gur EA (1999). Biomechanical analysis of the orbitozygomatic complex in human cadavers: examination oad sharing and failure patterns after fixation with titanium and bioresorbable systems. J Craniofac Surg.

[7] Rohner D, Tay A, Meng CS (2002). The sphenozygomatic suture as a key site for osteosynthesis of the orbitozygomatic complex in panfacial fractures: a biomechanical studying human cadavers based on clinical practice. Plast Reconstr Surg.

[8] Deveci M, Eski M, Gurses S, Yucesoy CA, Selmanpakoglu N, Akkas N. Biomechanical analysis of the rigid fixation of zygoma fractures: an experimental study. J Craniofacial Surg. 2004;15(4):595–602. 10.1097/00001665-200407000-00013 15213537

[9] pley BL (2000). Zygomaticomaxillary fracture repair with resorbable plates and screws. J Craniofac Surg.

[10] Hanemann Jr M, Simmons O, Jain S, Baratta R, Guerra AB, Metzinger SE (2005). A comparison of combinations of titanium and resorbable plating systems for repair of isolated zygomatic fractures in the adult: a quantitative biomechanical study. Ann Plast Surg.

[11] Ehrenfeld M, Manson PN, Prein J. Principles of internal fixation of the craniomaxillofacial skeleton: trauma and orthognathic surgery. 2012.

[12] Davidson J, Nickerson D, Nickerson B (1990). Zygomatic fractures: comparison of methods of internal fixation. Plast Reconstr Surg.

[13] Hwang K (2010). One-point fixation of tripod fractures of zygoma through a lateral brow incision. J Craniofac Surg.

[14] Kim ST, Go DH, Jung JH, Cha HE, Woo JH, Kang IG (2011). Comparison of 1-point fixation with 2-point fixation in treating tripod fractures of the zygoma. J Oral Maxillofac Surg.

[15] Kim JH, Lee JH, Hong SM, Park CH (2012). The effectiveness of 1-point fixation for zygomaticomaxillary complex fractures. Arch otorhinolaryngol - head neck surg.

[16] Rana M, Warraich R, Tahir S, Iqbal A, von See C, Eckardt AM (2012). Surgical treatment of zygomatic bone fracture using two points fixation versus three point fixation-a randomised prospective clinical trial. Trials.

[17] Nasr WF, ElSheikh E, El-Anwar MW, Sweed AH, Bessar A, Ezzeldin N (2018). Two-versus three-point internal fixation of displaced zygomaticomaxillary complex fractures. Craniomaxillofac Trauma Reconstr.

[18] Neto RM, Zotarelli-Filho IJ, Ribeiro da Silva CE (2023). Meta-analysis of the Major Clinical results of the treatment with 1-Point fixation in Fractures in the zygomatic-maxillary complex: success rate and Complications. J Oral Maxillofac Surg.

[19] Yang S, Cho JY, Shim WC, Kim S (2021). Retrospective study about the postoperative stability of zygomaticomaxillary complex fracture. Maxillofac Plast Reconstr Surg.

[20] Dal Santo F, Ellis E, Throckmorton GS (1992). The effects of zygomatic complex fracture on masseteric muscle force. J Oral Maxillofac Surg.

[21] Ellis IIIE, Perez D (2014). An algorithm for the treatment of isolated zygomatico-orbital fractures. J Oral Maxillofac Surg.

[22] Kim H-J, Bang K-H, Park E-J, Cho Y-C, Sung I-Y, Son J-H (2018). Evaluation of postoperative stability after open reduction and internal fixation of zygomaticomaxillary complex fractures using cone beam computed tomography analysis. J Craniofac Surg.

[23] Fujioka M, Yamanoto T, Miyazato O, Nishimura G (2002). Stability of one-plate fixation for zygomatic bone fracture. Plast Reconstr Surg.

[24] Al-Qattan MM, Gelidan A (2021). Fixation at the Inferior Orbital Rim in Medially rotated zygomatic complex fractures. Plast Reconstr Surg - Glob Open.

[25] Fallahi HR, Keyhan SO, Forooghi I, Mahoutchi DS, Abdollahi MR (2020). The effects of different plate fixation methods in the zygomaticomaxillary complex: a finite element analysis. J Oral Maxillofac Surg Med Pathol.

[26] Inehart GC, Marsh JL, Hemmer KM, Bresina S (1989). Internal fixation of malar fractures: an experimental biophysical study. Plast Reconstr Surg.

[27] Fernández JR, Gallas M, Burguera M, Viano J (2003). A three-dimensional numerical simulation of mandible fracture reduction with screwed miniplates. J Biomech.

[28] Cox T, Kohn MW, Impelluso T (2003). Computerized analysis of resorbable polymer plates and screws for the rigid fixation of mandibular angle fractures. J Oral Maxillofac Surg.

[29] Yuan-Kun T, Yau-Chia L, Wen-Jen Y, Li-Wen C, You-Yao H, Yung-Chuan C, editors. Temperature rise simulation during a Kirschner pin drilling in bone. Bioinformatics and biomedical engineering, 2009. ICBBE 2009. 3rd International Conference on; 2009.

[30] Osman RB, Swain MV (2015). A critical review of dental implant materials with an emphasis on titanium versus zirconia. Materials.

[31] Wang D, Roy A, Silberschmidt VV (2016). Hybrid cutting of bio-tissues. Procedia CIRP.

[32] Chancharoen S, Santiwong P, Seriwatanachai D, Khantachawana A, Chintavalakorn R (2022). A novel alveolar distractor incorporating nickel–Titanium Alloy springs: a preliminary in Vitro Study. Materials.

[33] Foletti JM, Martinez V, Haen P, Godio-Raboutet Y, Guyot L, Thollon L (2019). Finite element analysis of the human orbit. Behavior of titanium mesh for orbital floor reconstruction in case of trauma recurrence. J Stomatology Oral Maxillofacial Surg.

[34] Dos Santos MV, Elias CN, Cavalcanti Lima JH (2011). The effects of superficial roughness and design on the primary stability of dental implants. Clin Implant Dent Relat Res.

[35] Ben Nissan B (1987). Three dimensional modelling and finite element distortion analysis of the mandible.

[36] Sugiura T, Horiuchi K, Sugimura M, Tsutsumi S (2000). Evaluation of threshold stress for bone resorption around screws based on in vivo strain measurement of miniplate. J Musculoskelet Neuronal Interact.

[37] Shyam Sundar S, Nandlal B, Saikrishna D, Mallesh G (2012). Finite element analysis: a maxillofacial surgeon’s perspective. J Oral Maxillofac Surg.

[38] Szwedowski TD, Whyne CM, Fialkov JA (2010). Toward characterization of craniofacial biomechanics. J Craniofac Surg.

[39] Fernàndez E, Gil F, Aparicio C, Nilsson M, Sarda S, Manero J (2003). Materials in dental implantology.

[40] Hart NH, Nimphius S, Rantalainen T, Ireland A, Siafarikas A, Newton RU (2017). Mechanical basis of bone strength: influence of bone material, bone structure and muscle action. J Musculoskelet Neuronal Interact.

[41] Çimen E, Önder ME, Cambazoglu M, Birant E (2016). Comparison of different fixation types used in unilateral mandibular condylar fractures: an in vivo study with new biomechanical model. J Craniofac Surg.

[42] Prado FB, Freire AR, Cláudia Rossi A, Ledogar JA, Smith AL, Dechow PC, Strait DS, Voigt T, Ross CF (2016). Review of in vivo bone strain studies and finite element models of the zygomatic complex in humans and nonhuman primates: implications for clinical research and practice. Anat Rec.

